# From Genotype to Phenotype: Investigating *SLC22A5* Variants and Their Significance in Carnitine Deficiency: A Systematic Review Study

**DOI:** 10.1111/jcmm.71273

**Published:** 2026-07-21

**Authors:** Amir Ghaffari Jolfayi, Mahdieh Soveizi, Niloofar Naderi, Amirali Soheili, Maryam Pourirahim, Leyla Abdolkarimi, Majid Maleki, Samira Kalayinia

**Affiliations:** ^1^ Cardiovascular Research Center Rajaie Cardiovascular Institute Tehran Iran; ^2^ Cardiogenetic Research Center Rajaie Cardiovascular Institute Tehran Iran

**Keywords:** cardiomyopathy, mutation, OCTN2, primary carnitine deficiency, SLC22A5

## Abstract

Primary carnitine deficiency (PCD) is an autosomal recessive disorder caused by mutations in the *SLC22A5* gene, which encodes the organic cation transporter 2 (OCTN2). These mutations impair carnitine transport and fatty acid metabolism, leading to a wide range of clinical symptoms, from mild fatigue to severe cardiomyopathy. Although over 100 mutations have been identified, the correlation between specific genotypes and pathogenicity remains unclear, necessitating further investigation into their molecular and clinical impacts. This systematic review aims to evaluate the association between *SLC22A5* mutations and PCD, with a focus on cardiomyopathy, to characterise the molecular and functional consequences of these variants, and to explore their implications for diagnosis, treatment, and personalised care. A systematic search of PubMed was conducted, focusing on studies reporting genetic, clinical, and biochemical data related to SLC22A5. Variants were classified according to ACMG guidelines and CADD scoring, and the data were synthesised through descriptive analyses. Most reported variants are missense mutations, accounting for approximately 80% of cases, followed by deletions (12.7%), while duplications, insertions, and deletion–insertion variants are rare. According to ACMG classification, the majority of variants are pathogenic or likely pathogenic (≈72%), with VUS comprising about 24%, and benign variants being uncommon. Exons 1 and 8 have the highest frequencies, whereas Exons 5 and 6, despite fewer variants, exhibit higher mean CADD scores, suggesting greater functional impact. The findings revealed that Exons 1 and 8 harboured the most frequent mutations, while Exons 5 and 6 showed the highest pathogenicity. Nonsense variants exhibit the highest pathogenicity and CADD scores, followed by frameshift and missense mutations. Severe truncating mutations were linked to early‐onset cardiomyopathy. Intronic variants are infrequent but include sites with high CADD scores, indicating potential regulatory relevance. Overall, these findings demonstrate that variant pathogenicity is influenced more by functional impact than by frequency alone.

## 1 Introduction

Carnitine deficiency, a condition characterised by insufficient levels of carnitine in the body, has been associated with a spectrum of clinical manifestations, ranging from mild fatigue to severe cardiomyopathy [1, 2]. The intricate regulation of carnitine uptake and distribution within the body involves several key genes, among which *SLC22A5* stands out as a pivotal player. This gene encodes the organic cation transporter 2 (OCTN2), which is primarily responsible for transporting carnitine across cell membranes, thereby maintaining cellular carnitine homeostasis [3, 4]. Variants within the *SLC22A5* gene have been linked to alterations in carnitine transport efficiency, resulting in disruptions in carnitine metabolism and the subsequent manifestation of carnitine deficiency syndromes. Of particular interest is the association between *SLC22A5* variants and the development of cardiomyopathy, a serious complication of carnitine deficiency characterised by impaired cardiac function [4, 5, 6]. Despite significant advancements in our understanding of carnitine deficiency and its genetic underpinnings, a comprehensive review focusing specifically on the relationship between *SLC22A5* variants and carnitine deficiency‐associated cardiomyopathy is lacking. Such a review is essential not only for consolidating existing knowledge but also for identifying gaps in understanding and avenues for future research. In this systematic review, we aim to explore the current literature on *SLC22A5* variants and their association with carnitine deficiency and cardiomyopathy. This study aims to systematically map the human genetic landscape of SLC22A5 (OCTN2) in primary carnitine deficiency by compiling and standardizing all reported variants and evaluating the strength of supporting evidence, ACMG classification, available functional assays, and CADD as supportive where applicable; in doing so, it provides curated variant‐to‐phenotype tables with explicit provenance, analyses the distribution of variant consequences across the gene, clarifies genotype–phenotype patterns and sources of heterogeneity, and identifying gaps to guide future research.

## 2 Methods and Materials

### 2.1 Literature Search Strategy

A systematic search of PubMed was conducted to identify studies investigating the association between *SLC22A5* variants and carnitine deficiency. The search strategy was designed to encompass key terms related to carnitine deficiency and the *SLC22A5* gene. The query used for the search included variations of terms related to carnitine deficiency and *SLC22A5*:

Pubmed search syntax: (((carnitine deficiency [Title/Abstract]) OR (primary carnitine deficiency [Title/Abstract])) OR (PCD[Title/Abstract])) OR Systemic carnitine deficiency AND ((((SLC22A5 protein, human) OR (SLC22A5)) OR (OCTN2)) OR (solute carrier family 22 member 5)) OR (organic cation transporter novel family member 2).

Scopus search syntax ((TITLE‐ABS‐KEY (slc22a5 protein human) OR TITLE‐ABS‐KEY (slc22a5 gene) OR TITLE‐ABS‐KEY (solute carrier family 22 member 5) OR TITLE‐ABS‐KEY (OCTN2) OR TITLE‐ABS‐KEY (organic cation transporter novel family member 2))) AND ((TITLE‐ABS‐KEY (carnitine deficiency) OR TITLE‐ABS‐KEY (primary carnitine deficiency) OR TITLE‐ABS‐KEY (PCD) OR TITLE‐ABS‐KEY (Systemic carnitine deficiency))).

To minimise the risk of missing records, we conducted backward/forward citation tracking of all eligible records and manually searched the reference lists of gene‐focused reviews and index papers.

### 2.2 Study Selection and Inclusion Criteria

*Population/Condition*: Humans with primary carnitine deficiency or related phenotypes in whom SLC22A5 variants were reported.

*Evidence types*: Primary studies reporting genetic findings (sequencing or genotyping) with clinical and/or biochemical data, and/or functional assays relevant to OCTN2.

*Outcomes*: Any clinical phenotype (e.g., cardiomyopathy, hypoglycemia), and/or functional transport data attributable to SLC22A5 variants.

*Inclusions*: Case reports, case series, and cohort descriptions.

*Exclusions*: Reviews, editorials, conference abstracts without primary data; animal‐only or cell‐only studies without human genotype context; non‐English full texts.

### 2.3 Data Extraction

Two independent reviewers conducted the literature search and screened the titles and abstracts of identified studies for relevance. Full‐text articles of potentially relevant studies were then assessed for eligibility based on the inclusion criteria. Data extraction was performed using a standardised form, which included the study name and variants reported. *SLC22A5* variants identified for further evaluation are considered.

### 2.4 Data Synthesis and Analysis

A narrative synthesis approach was employed to summarise the findings of the included studies. Key findings regarding the association between *SLC22A5* variants and carnitine deficiency‐related cardiomyopathy were synthesised and presented descriptively. We planned a narrative/descriptive synthesis a priori. A pooled meta‐analysis was not undertaken due to heterogeneity in study designs, outcomes, and the potential for overlapping cohorts, which precluded the valid aggregation of effect sizes.

### 2.5 Determining the Characteristics of Variants

Based on the ACMG and CADD scoring systems and the type of mutation, the features of the variants are identified and described below.

Variant classification was performed according to ACM guidelines, integrating computational predictions (CADD) and population frequency data. Functional validation results were included when available, although such data were limited and inconsistently reported across studies.

### 2.6 ACMG Score

The ACMG score, established by the American College of Medical Genetics and Genomics, provides essential guidelines for interpreting sequence variants, especially in the context of Mendelian disorders. These guidelines have been adapted to recommend standardised terms like “pathogenic” and “benign” for characterizing variants, ensuring consistency and clarity in variant classification. Moreover, the recommendation outlines a systematic approach that relies on diverse types of evidence, including population data, computational analysis utilizing predictive tools for missense and splice site prediction, functional data, and segregation data. This comprehensive approach ensures a thorough evaluation of variants, facilitating accurate classification into categories such as “pathogenic,” “likely pathogenic,” “uncertain significance,” “likely benign,” and “benign” based on specific criteria involving the strength and combination of evidence [7, 8]. A variant is considered likely pathogenic if it meets particular criteria, including the presence of one robust criterion (PVS1) along with one moderate criterion (PM1‐PM6). Alternatively, it can be classified as likely pathogenic if it demonstrates one strong criterion (PS1‐PS4) alongside one to two moderate criteria (PM1‐PM6), or if it possesses one strong criterion (PS1‐PS4) and at least two supporting criteria (PP1‐PP5). Additionally, variants may be designated as likely pathogenic if they satisfy the requirement of having three or more moderate criteria (PM1‐PM6). Moreover, a variant is classified as likely pathogenic if it meets specific combinations of moderate and supporting criteria, such as having two moderate criteria (PM1‐PM6) and at least two supporting criteria (PP1‐PP5), or exhibiting one moderate criterion (PM1‐PM6) along with at least four supporting criteria (PP1‐PP5).

Additional details are supplemented in “*Standards and guidelines for the interpretation of sequence variants: a joint consensus recommendation of the American College of Medical Genetics and Genomics and the Association for Molecular Pathology*” [8].

### 2.7 CADD Score

The CADD score (Combined Annotation Dependent Depletion) is a versatile tool for evaluating the pathogenicity of genetic variants in the human genome. Its framework compares variants that have undergone natural selection with simulated mutations, providing robust correlations with allelic diversity, pathogenicity, and regulatory effects of coding and non‐coding variants. Notably, C‐scores for variants associated with complex traits in genome‐wide association studies (GWAS) are significantly higher than those of matched controls, indicating improved accuracy in larger studies. CADD uses a machine‐learning model to distinguish simulated de novo variants from those that have persisted in human populations since the split from chimpanzees. The efficacy of this tool is further bolstered by features derived from the ESM‐1v protein language model for variant coding and from a convolutional neural network trained on open chromatin regions for regulatory variants [9, 10, 11, 12].

### 2.8 Type of Mutation

The type of mutation is evaluated in the two concepts of “evaluating the structure of the final protein product” based on the genetic alteration and assessing the alteration in the genetic code.

### 2.9 Risk of Bias Assessment

Because the included evidence comprised primarily case reports/series and functional assays, commonly used RoB tools for comparative designs were not applicable. We therefore did not perform a formal RoB assessment. To contextualise evidence strength, we prioritised variant‐level indicators standard in clinical genetics (ACMG classification, CADD scoring, and whether functional validation was reported).

### 2.10 Ethical Considerations

This review utilised only data from previously published studies, and no human subjects were directly involved in this research. Therefore, ethical approval was not required.

## 3 Results

### 3.1 Carnitine Metabolism

Carnitine is a hydrophilic quaternary amine essential for metabolism, primarily responsible for transporting long‐chain fatty acids from the cytosol to the mitochondria for β‐oxidation. While carnitine can be synthesised in the body, most of it is obtained from dietary sources, particularly animal products. It is not metabolised and is excreted in urine, with homeostasis maintained through efficient renal reabsorption that balances dietary intake [13]. In non‐vegetarians, approximately 75% of carnitine is obtained from dietary sources, while around 25% is produced endogenously. Carnitine homeostasis is maintained by renal reabsorption, dietary intake, and endogenous synthesis. The biosynthesis of carnitine requires two key amino acid precursors, lysine and methionine, and involves four enzymes [14].

### 3.2 Carnitine Metabolism Errors

There are two primary groups of carnitine metabolism disorders: First, defects in carnitine biosynthesis, and second, carnitine transport. In carnitine transport defects, there is significant carnitine depletion, leading to symptoms such as metabolic issues in infancy, cardiomyopathy in childhood, and fatigability in adulthood. Some individuals may remain asymptomatic. These symptoms arise from inadequate carnitine availability, which impairs fatty acid β‐oxidation [13, 15]. In contrast, carnitine biosynthesis disorders do not typically cause carnitine deficiency because dietary intake and renal reabsorption are usually sufficient to maintain normal levels. However, these biosynthetic defects can still lead to pathological conditions due to imbalances in intermediate metabolites or carnitine deficiency during critical developmental periods, such as early life, when the brain and other organs are maturing [13]. Additionally, various metabolic diseases and other medical conditions can cause excessive carnitine loss, resulting in secondary carnitine deficiency.

### 3.3 OCTN2 as Carnitine Transporter Protein

OCTN2 is responsible for transporting carnitine into cells. Carnitine plays a critical role in transporting long‐chain fatty acids from the cytosol to the mitochondrial matrix for β‐oxidation. This process is facilitated by the mitochondrial carnitine‐acylcarnitine cycle, which involves three key enzymes: carnitine palmitoyltransferase I (CPT I), carnitine‐acylcarnitine translocase (CACT), and carnitine palmitoyltransferase II (CPT II). Together, these enzymes shuttle fatty acids across the mitochondrial membrane, enabling energy production through β‐oxidation [14]. OCTN2 consists of 12 transmembrane domains, with six extracellular loops and five intracellular loops. It also features intracellular N‐ and C‐termini, making up a total of 25 distinct topological regions [16].

### 3.4 *SLC22A5* Gene: Location and Role

The *SLC22A5* gene, located on chromosome 5q31 and known as OCTN2 or CDSP, encodes a crucial plasma membrane protein. This protein serves dual roles: it transports organic cations and acts as a sodium‐dependent, high‐affinity carnitine transporter. Its primary function is to facilitate the active cellular uptake of carnitine. Mutations in the *SLC22A5* gene lead to systemic PCD, an autosomal recessive disorder. The gene is broadly expressed across tissues, with particularly high expression in the kidney and small intestine; alternative splicing generates multiple transcript variants [17].

### 3.5 Primary Carnitine Deficiency Clinical Manifestations

Systemic PCD is a rare autosomal recessive disorder that disrupts carnitine transportation, leading to a wide range of clinical manifestations. The condition presents with a broad spectrum of symptoms, varying in severity, age, and organ involvement. In infants, it commonly manifests as hypoketotic hypoglycemia, hepatomegaly, raised liver enzymes, and elevated ammonia levels. As children, individuals may experience muscle weakness, elevated creatine kinase (CK), and cardiomyopathy, while in adults, PCD often leads to cardiomyopathy, arrhythmia, and chronic fatigue [18].

### 3.6 Laboratory Findings of PCD

PCD can initially be suspected through newborn screening. Still, a definitive diagnosis is established by identifying low plasma free carnitine levels (< 5 μM, compared with the normal range of 25–50 μM), reduced carnitine transport in fibroblasts (less than 10% of control values), and confirming mutations in the *SLC22A5* gene. This gene encodes a sodium‐dependent transporter that transports carnitine into cells, which is vital for energy production via fatty acid oxidation, particularly in the heart [18].

### 3.7 Primary vs. Secondary Carnitine Deficiency

Primary or secondary carnitine deficiency occurs in various clinical conditions, except for carnitine palmitoyltransferase type I deficiency and the classic adult form of carnitine palmitoyltransferase type II deficiency. The only known form of primary carnitine deficiency results from a genetic defect that impairs carnitine transport across the cell membrane. This condition responds significantly to oral carnitine therapy. In contrast, secondary carnitine deficiencies show a less pronounced response to carnitine supplementation. Management of secondary deficiencies typically involves a high‐carbohydrate, low‐fat diet with frequent meals and vitamin or cofactor supplementation (such as carnitine, glycine, and riboflavin). In cases involving inborn errors of the carnitine cycle or long‐chain fatty acid oxidation, medium‐chain triglycerides may offer dietary benefits [19].

Among the mutations that have been identified in the *SLC22A5* gene, the c.136C>T (p.P46S) mutation is the most common. It's essential to differentiate primary carnitine deficiency (PCD) from secondary carnitine deficiencies, which can arise from conditions such as organic acidemias and fatty acid oxidation disorders. A thorough understanding of the genetic basis and clinical presentation of CDSP is crucial for early intervention and for preventing severe complications, particularly in the heart and skeletal muscles. Among the variants extracted from the previously published article, substitution mutations are the most prevalent, accounting for 80.29% of the total reported variants. Deletions are the second most common type, accounting for 12.7%. Other types of variants, including duplications and insertions, are relatively rare, each accounting for approximately 2%–3% of the total. Deletion/insertion variants are the least common, accounting for 1.4%.

### 3.8 Distribution Type and Outcome of *SLC22A5* Variants

**FIGURE 1:**
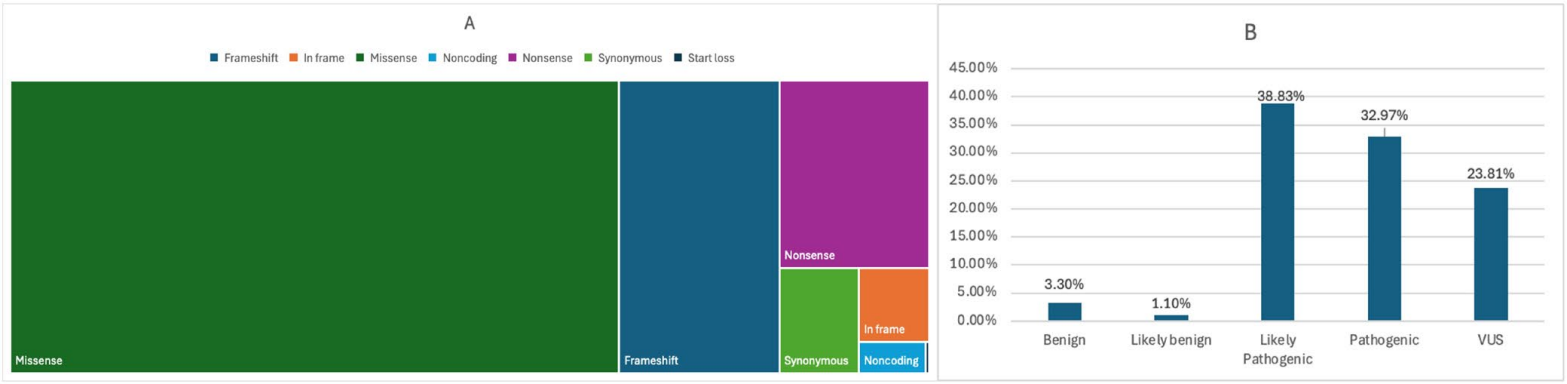
Distribution of the type of variants and their impact on the development of ideas in the previous literature. (A) Frequency of each type of variants (B) Pathogenicity of the variants.

**FIGURE 2:**
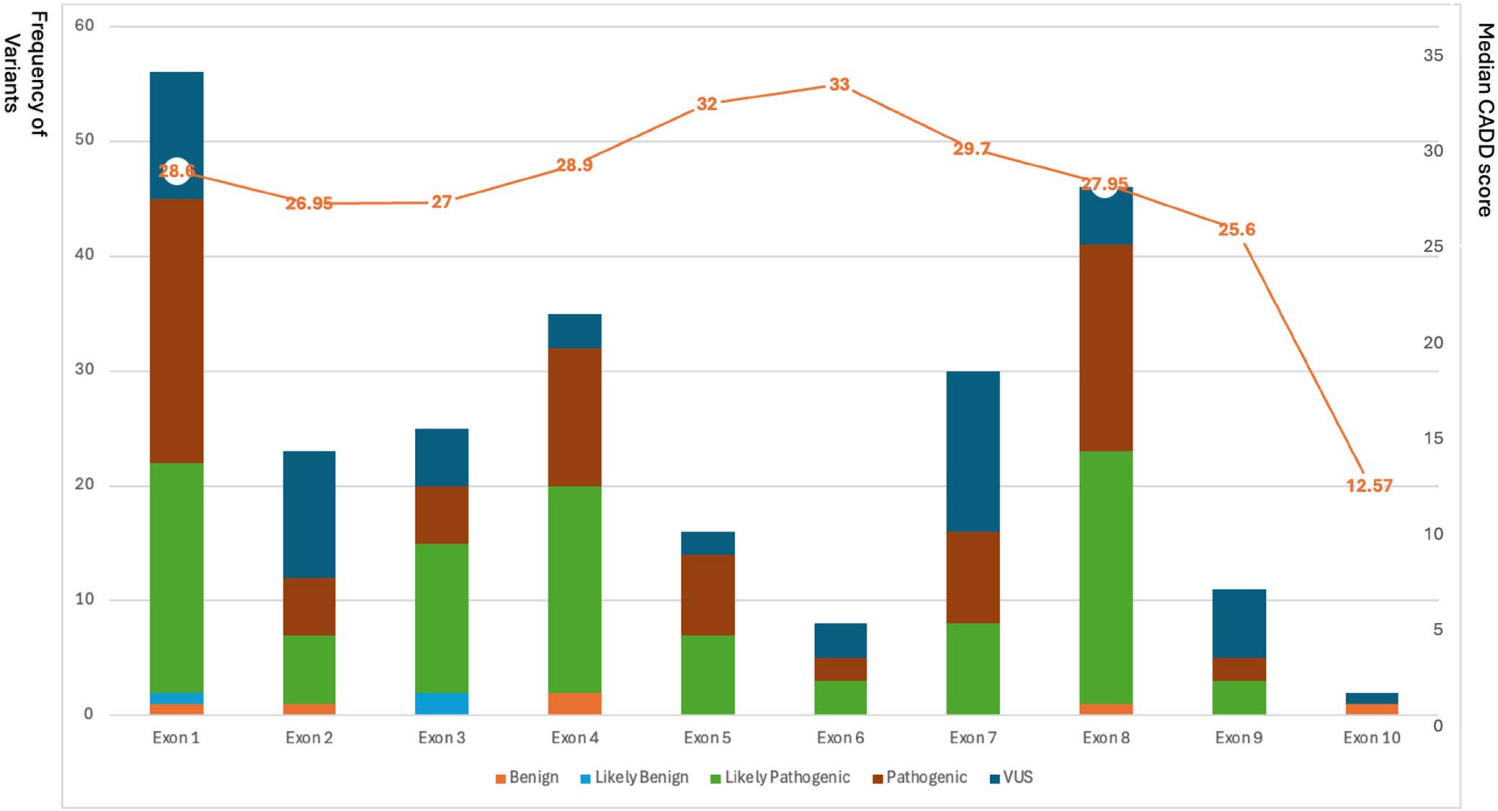
Distribution of the variants across the exons, their pathogenicity, and the attributable CADD score of each.

**FIGURE 3:**
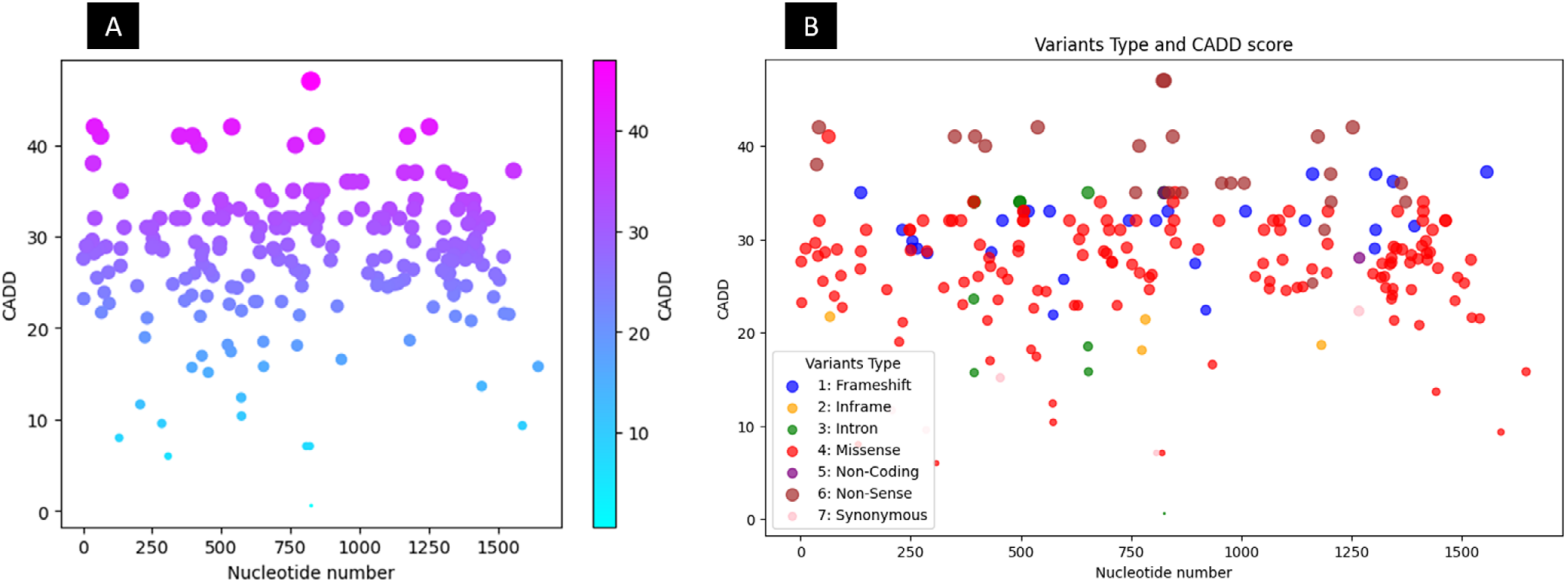
Distribution of the variants across the exon and their pathogenicity (Diameter of each variant, correlated with its CADD score). (A) Comparison of the CADD score relative to the nucleotide number across the gene. (B) Predictive CADD score for each variant type across the gene.

In terms of pathogenicity, as classified by the ACMG, likely pathogenic and pathogenic variants predominate, accounting for 38.83% and 32.97%, respectively. Variants of uncertain significance (VUS) represent 23.81%, indicating a moderate level of uncertainty in clinical interpretation. Benign and likely benign variants account for 3.3% and 1.1%, respectively, suggesting that most identified variants are likely to have clinical significance (Figure 1 and Table 1) reveals key insights. Among exons, Exon 1 has the highest variant frequency (21.62%), with a mean CADD (Combined Annotation‐Dependent Depletion) score of 27.24, indicating a high likelihood of functional impact. Exon 8 follows closely, with 17.76% of exon variants and a mean CADD score of 27.61. Exons 5 and 6, although at lower frequencies (6.18% and 3.09%), exhibit higher mean CADD scores (30.61 and 32.20), suggesting these positions may carry more deleterious effects despite their lower variant frequencies (Figure 2). Introns have a relatively low variant count, with Intron 1 having the highest frequency at 1.93% and a mean CADD score of 28.26, indicating substantial functional importance. Introns 3 and 4 follow with frequencies of 1.16% and 1.54% of all variants, and mean CADD scores of 23.10 and 26.39, respectively. Introns with fewer variants, like Introns 2 and 7, show higher mean CADD scores of 34 and 28, potentially highlighting specific functional impacts despite their low occurrence. Additionally, two variants in the untranslated region (UTR) have a lower mean CADD score of 7.73, suggesting a reduced functional impact relative to coding regions. Overall, the mean CADD scores across exons and introns indicate that variant impact is not solely determined by frequency, with specific low‐frequency variants potentially carrying significant functional consequences. As demonstrated in Figure 3, Nonsense variants exhibit the highest pathogenicity and CADD scores, whereas frame‐shift and missense variants are ranked in the following order (Tables 2 and 3).

**TABLE 1:** Distribution type and impact of *SLC22A5* variants.

*Type of variant*	
Deletion	35 (12.7%)
Duplication	7 (2.55%)
Insertion	8 (2.92%)
Substitution	220 (80.29%)
Deletion/Insertion	4 (1.46%)
*Impact of the variant*	
Frameshift	45 (17.37%)
In frame	5 (1.93%)
Missense	171 (66.02%)
Noncoding	2 (0.77%)
Nonsense	27 (10.42%)
Synonymous	8 (3.09%)
Start loss	1 (0.039%)
*ACMG classification of the reported variants*	
Benign	9 (3.3%)
Likely benign	3 (1.1%)
Likely Pathogenic	106 (38.83%)
Pathogenic	90 (32.97%)
VUS	65 (23.81%)

**TABLE 2:** Distribution of the variant through the exons and introns.

Location	Frequency (%)	CADD score, median [IQR]
Exon 1	56 (20.7%)	28.60 [25.35–31.85]
Exon 2	24 (8.9%)	26.95 [24.40–29.20]
Exon 3	25 (9.3%)	27.00 [22.60–31.70]
Exon 4	35 (13.0%)	28.90 [26.20–31.95]
Exon 5	16 (5.9%)	32.00 [28.76–35.24]
Exon 6	8 (3.0%)	33.00 [29.60–36.40]
Exon 7	30 (11.1%)	29.70 [26.20–33.20]
Exon 8	46 (17.0%)	27.95 [25.00–30.63]
Exon 9	11 (4.1%)	25.60 [21.68–29.53]
Exon 10	2 (0.7%)	12.57 [10.95–14.19]
Intron 1	5 (1.9%)	34.00 [28.80–39.20]
Intron 2	1 (0.4%)	34.00 [34.00–34.00]
Intron 3	3 (1.1%)	18.51 [13.71–23.31]
Intron 4	4 (1.5%)	35.00 [30.70–39.30]
Intron 7	1 (0.4%)	28.00 [28.00–28.00]
5′ UTR	2 (0.7%)	7.74 [7.67–7.81]

Abbreviation: UTR, untranslated region.

**TABLE 3:** Detailed variant types with nucleotide and amino acid changes, CADD scores, and ACMG classification.

Nucleotide change	Amino acid change	dbSNP	CADD	ACMG	Intron/Exon	Mutation type	References
c.254_264dup	p.Ile89Glyfs*45	rs377767449	29.8	P	Exon 1	Frameshift	[6, 20, 21]
c.338G>A	p.Cys113Tyr	rs727504159	32	P	Exon 1	Missense	[20, 22, 23, 24, 25, 26, 27, 28, 29]
c.1088T>C	p.Leu363Pro	rs386134214	31	LP	Exon 7	Missense	[30]
c.1340A>C	p.Gly12Ser;Tyr447Ser	—	28	LP	Exon 8	Missense	[30]
c.149G>A	p.Cys50Tyr	—	31	LP	Exon 1	Missense	[30]
c.232C>T	p.Pro78Ser	rs1485828747	21.1	VUS	Exon 1	Missense	[31]
c.457G>C	p.Val153Leu	—	26.4	VUS	Exon 2	Missense	[30]
c.632A>G	p.Tyr211Cys	rs121908888	30	P	Exon 3	Missense	[26, 27, 32, 33]
c.695C>T	p.Thr232Met	rs114269482	32	P	Exon 4	Missense	[21, 27, 28, 30, 34, 35]
c.825–1G>C	—	rs1057516805	35	P	Intron 4	Non coding	[30]
c.680G>A	p.Arg227His	rs185551386	34	P	Exon 4	Missense	[20, 27, 36, 37, 38]
c.1006C>T	p.Arg336Ter	rs754008420	36	P	Exon 6	Nonsense	[39]
c.1031C>T	p.Thr344Ile	—	26	VUS	Exon 6	Missense	[40]
c.1049T>C	p.Leu350Pro	—	27.4	VUS	Exon 6	Missense	[41]
c.1051T>C	p.Trp351Arg	rs68018207	31	VUS	Exon 6	Missense	[6, 21, 35, 42]
c.1266A>G	p.Pro422Pro	rs1162414161	22.3	VUS	Exon 7	Synonymous	[43]
c.1063T>C	p.Ser355Pro	—	24.7	LP	Exon 7	Missense	[43]
c.1064C>T	p.Ser355Leu	rs1385634398	25.5	VUS	Exon 7	Missense	[20, 27]
c.1072T>A	p.Tyr358Asn	rs61731073	32	P	Exon 7	Missense	[27]
c.1085C>T	p.Ser362Leu	rs886042092	32	VUS	Exon 7	Missense	[44, 45]
c.1093A>C	p.Thr365Pro	rs1382542971	27.8	VUS	Exon 7	Missense	[46]
c.1101C>G	p.Asn367Lys	—	24.5	VUS	Exon 7	Missense	[30]
c.1108G>A	p.Gly370Arg	—	33	VUS	Exon 7	Missense	[47, 48]
c.1126T>C	p.Cys376Arg	—	24.8	VUS	Exon 7	Missense	[49]
c.1139C>T	p.Ala380Val	rs746187344	24.9	LP	Exon 7	Missense	[27, 34, 49, 50, 51]
c.1144_1162del	p.Val382CysfsTer45	—	32	LP	Exon 7	Frameshift	[27, 34, 50, 51]
c.1160A>G	p.Tyr387Cys	—	26.8	VUS	Exon 7	Missense	[27, 34, 51]
c.1161_1162insA	p.Val388SerfsTer135	—	37	LP	Exon 7	Frameshift	[27, 30, 38]
c.1161T>G	p.Tyr387Ter	rs72552731	25.3	LP	Exon 7	Nonsense	[24, 27, 34, 44, 50]
c.1173G>A	p.Trp391Ter	rs1056070920	41	LP	Exon 7	Nonsense	[27, 30, 38]
c.1181_1183del	p.Leu394del	rs386134215	18.66	P	Exon 7	Frameshift	[52]
c.1188T>G	p.Tyr396Ter	rs1057519051	31	P	Exon 7	Nonsense	[6, 53]
c.1193C>T	p.Pro398Leu	rs144547521	26.4	P	Exon 7	Missense	[21, 27, 54, 55, 56]
c.1195C>T	p.Arg399Trp	rs267607054	29.5	P	Exon 7	Missense	[6, 26, 27, 30, 34, 38, 40, 46, 47, 48, 49, 50, 57, 58, 59, 60, 61, 62]
c.1196G>A	p.Arg399Gln	rs121908891	33	P	Exon 7	Missense	[6, 21, 27, 34, 47, 61, 62, 63]
c.1202_1203insA	p.Tyr401Ter	rs121908887	37	P	Exon 7	Nonsense	[6, 21, 35]
c.1203T>G	p.Tyr401Ter	—	34	LP	Exon 7	Nonsense	[64]
c.1252C>T	p.Gln418Ter	rs1057518297	42	P	Exon 7	Nonsense	[28, 49, 65]
c.1267+3_1267+23del	—	rs386134216	28	VUS	Intron 7	Non coding	[6, 21]
c.1298T>C	p.Met433Thr	rs779385095	26.3	P	Exon 8	Missense	[48, 50, 66]
c.12C>G	p.Tyr4Ter	rs72552722	29	P	Exon 1	Missense	[6, 21, 35, 55, 67]
c.1302delG	p.Gly435fs*24	rs386134217	29	P	Exon 8	Frameshift	[6, 35]
c.1304_1313del10	p.Gly435Glufs*21	—	31	LP	Exon 8	Frameshift	[27]
c.1304del	p.Gly435AlafsTer24	rs386134217	37	P	Exon 8	Frameshift	[35]
c.1316T>G	p.Val439Gly	—	25.9	LP	Exon 8	Missense	[6]
c.1319C>T	p.Thr440Met	rs72552732	27.4	P	Exon 8	Missense	[6, 21, 27, 35, 55, 68, 69, 70]
c.131C>T	p.Ala44Val	rs199689597	8	P	Exon 1	Missense	[62, 71]
c.1324_1325delinsAT	p.Ala442Ile	rs267607053	26	P	Exon 8	Missense	[6, 27, 35, 60]
c.1327T>G	p.Phe443Val	—	24.8	LP	Exon 8	Missense	[27]
c.1336G>T	p.Val446Phe	rs72552733	27.3	P	Exon 8	Missense	[35, 62, 72]
c.1340A>G	p.Tyr447Cys	rs386134218	27.5	P	Exon 8	Missense	[6, 21, 35, 73]
c.1340A>T	p.Tyr447Phe	—	23.6	LP	Exon 8	Missense	[74]
c.1342G>T	p.Val448Leu	rs386134219	24	LP	Exon 8	Missense	[6, 21]
c.1343T>G	p.Val448Gly	—	24.7	LP	Exon 8	Missense	[27, 38]
c.1344del	p.Tyr449Thrfs*10	—	36.2	LP	Exon 8	Frameshift	[64]
c.1345T>G	p.Tyr449Asp	rs11568514	29.2	LP	Exon 8	Missense	[6, 27, 75, 76]
c.1346A>T	p.Tyr449Phe	—	21.3	LP	Exon 8	Missense	[73]
c.1351G>C	p.Ala451Pro	—	29	VUS	Exon 8	Missense	[77]
c.1354G>A	p.Glu452Lys	rs72552734	33	P	Exon 8	Missense	[6, 21, 27, 30, 62, 78, 79, 80]
c.1362T>G	p.Tyr454Ter	—	36	LP	Exon 8	Nonsense	[49]
c.1364C>G	p.Pro455Arg	rs1408166345	28.9	P	Exon 8	Missense	[27, 47]
c.136C>T	p.Pro46Ser	rs202088921	26.8	P	Exon 1	Missense	[21, 27, 30, 35, 62, 81, 82, 83]
c.137_159del	p.Pro46ArgfsTer84	—	35	LP	Exon 1	Frameshift	[27, 38]
c.1372delG	p.Val458Ter	—	34	LP	Exon 8	Nonsense	[59]
c.137C>T	p.Pro46Leu	rs377767445	28.7	P	Exon 1	Missense	[6]
c.1382T>G	p.Met461Arg	—	27.5	LP	Exon 8	Missense	[84]
c.1385G>A	p.Gly462Asp	rs2126791535	28.3	LP	Exon 8	Missense	[49]
c.1385G>T	p.Gly462Val	—	24.6	LP	Exon 8	Missense	[6]
c.1392_1409delinsCA	p.Val465Thrfs*29	rs386134220	31.4	LP	Exon 8	Frameshift	[6, 21]
c.1400C>G	p.Ser467Cys	rs60376624	27.9	P	Exon 8	Missense	[20, 37, 49, 74, 85]
c.1403C>G	p.Thr468Arg	rs386134221	20.8	P	Exon 8	Missense	[6, 21, 55, 86, 87, 88]
c.1409C>T	p.Ser470Phe	rs386134222	29.3	P	Exon 8	Missense	[87, 89]
c.1411C>T	p.Arg471Cys	rs749282641	32	P	Exon 8	Missense	[27, 34, 44, 57, 90]
c.1412G>C	p.Arg471Pro	rs386134223	34	P	Exon 8	Missense	[35]
c.1418G>T	p.Gly473Val	rs181483390	29.8	LP	Exon 8	Missense	[91]
c.1420A>C	p.Ser474Arg	—	28.5	LP	Exon 8	Missense	[38]
c.1421G>A	p.Ser474Asn	rs796052035	27.8	VUS	Exon 8	Missense	[30]
c.1427T>G	p.Leu476Arg	—	28.6	LP	Exon 8	Missense	[92]
c.1433C>T	p.Pro478Leu	rs72552735	31	P	Exon 8	Missense	[49, 93, 94, 95]
c.1441G>A	p.Val481Ile	rs11568513	13.65	B	Exon 8	Missense	[76]
c.1445A>G	p.Tyr482Cys	—	26.9	VUS	Exon 8	Missense	[49]
c.1462C>T	p.Arg488Cys	rs377216516	32	P	Exon 9	Missense	[6, 27, 46]
c.1463G>A	p.Arg488His	rs28383481	32	LP	Exon 9	Missense	[6, 21, 35, 55, 63, 96, 97, 98]
c.1484T>C	p.Met495Thr	—	23.4	VUS	Exon 9	Missense	[99]
c.1490G>A	p.Ser497Asn	—	25.9	VUS	Exon 9	Missense	[27, 38]
c.1505C>A	p.Thr502Lys	—	25.3	VUS	Exon 9	Missense	[49]
c.1520T>C	p.Leu507Ser	rs1157198543	27.8	LP	Exon 9	Missense	[27]
c.1522T>C	p.Phe508Leu	rs11568521	21.6	VUS	Exon 9	Missense	[76]
c.1540G>C	p.Gly514Arg	—	21.5	VUS	Exon 9	Missense	[45]
c.1556_1559dupACAC	p.Ile521HisfsTer3	rs386134225	37.2	P	Exon 9	Frameshift	[6, 21, 35]
c.1588A>G	p.Met530Val	rs11568524	9.331	VUS	Exon 10	Missense	[76]
c.1645C>T	p.Pro549Ser	rs11568525	15.8	B	Exon 10	Missense	[27, 76]
c.196A>C	p.Thr66Pro	rs1476076948	24.6	VUS	Exon 1	Missense	[27]
c.207G>C	p.Leu69Leu	rs377767446	11.65	VUS	Exon 1	Synonymous	[100]
c.224G>C	p.Arg75Pro	rs757711838	19	VUS	Exon 1	Missense	[27]
c.231_234del	p.Pro78ThrfsTer51	rs377767447	31	LP	Exon 1	Frameshift	[80]
c.248G>A	p.Arg83His	—	31	LP	Exon 1	Missense	[6, 20, 27, 35, 49, 59]
c.250T>A	p.Tyr84Asn	—	28.8	LP	Exon 1	Missense	[27, 34, 49, 50, 61]
c.252_265dup	p.Ile89ThrfsTer46	rs1554085973	29	P	Exon 1	Frameshift	[49]
c.265‐266ins GGCTCGCCACC	p.Ile89ArgfsTer45	rs386134189	29	LP	Exon 1	Frameshift	[99]
c.278C>T	p.Ser93Leu	rs386134190	32	VUS	Exon 1	Missense	[41]
c.285T>C	p.Leu95	rs2631365	9.561	B	Exon 1	Synonymous	[76]
c.287G>C	p.Gly96Ala	rs377767450	28.7	VUS	Exon 1	Missense	[27]
c.288delG	p.Leu97TrpfsTer33	—	28.5	LP	Exon 1	Frameshift	[50, 66, 91]
c.2T>G	p.Met1?	rs1554085885	27.6	LP	Exon 1	Missense	[81]
c.308T>G	p.Val103Gly	—	6	LP	Exon 1	Missense	[26, 27, 49]
c.325G>A	p.Glu109Lys	—	24.8	VUS	Exon 1	Missense	[101]
c.344A>G	p.Asp115Gly	rs386134192	32	VUS	Exon 1	Missense	[6, 21]
c.34G>A	p.Gly12Ser	rs139203363	29.6	LP	Exon 1	Missense	[27, 102]
c.350G>A	p.Trp117Ter	—	41	P	Exon 1	Nonsense	[27]
c.364G>T	p.Asp122Tyr	rs201082652	32	P	Exon 1	Missense	[27]
c.368T>G	p.Val123Gly	rs748605096	23	VUS	Exon 1	Missense	[27]
c.371A>G	p.Tyr124Cys	rs762126547	25.4	LP	Exon 1	Missense	[49]
c.37G>T	p.Glu13Ter	—	38	P	Exon 1	Nonsense	[103]
c.393+5G>A	—	rs1554086029	23.6	VUS	Intron 1	Intron	[59]
c.393G>C	p.Glu131Asp	—	34	VUS	Exon 1	Missense	[6]
c.394‐1G>A	—	—	34	LP	Intron 1	Intron	[27, 34, 50, 51]
c.394‐1G>T	—	rs1057517106	34	P	Intron 1	Intron	[49, 65, 99]
c.396G>A	p.Trp132Ter	rs72552727	41	P	Exon 2	Nonsense	[20, 27, 59, 77, 104]
c.3G>T	p.Met1Leu	rs121908892	23.2	LP	Exon 1	Missense	[6, 21, 35, 86]
c.403G>A	p.Val135Met	—	26	VUS	Exon 2	Missense	[45]
c.407G>A	p.Cys136Tyr	rs1266213552	29.4	VUS	Exon 2	Missense	[20]
c.40T>A	p.Trp14Arg	rs756863825	28.2	P	Exon 1	Missense	[49]
c.419G>A	p.Trp140Ter	rs796052039	40	P	Exon 2	Nonsense	[6]
c.428C>T	p.Pro143Leu	rs1178584184	28	P	Exon 2	Missense	[20, 22, 26, 27, 38, 47, 49, 50, 65, 85, 95]
c.42G>A	p.Trp14Ter	rs796052036	42	P	Exon 1	Nonsense	[49]
c.430C>T	p.Leu144Phe	rs10040427	16.98	B	Exon 2	Missense	[76, 96]
c.431T>C	p.Leu144Pro	—	27.1	VUS	Exon 2	Missense	[41, 47, 49, 99]
c.433dupA	p.Thr145Asnfs*50	—	28.6	LP	Exon 2	Frameshift	[59]
c.43G>T	p.Gly15Trp	rs267607052	32	P	Exon 1	Missense	[6, 21, 27, 35, 49, 60, 62]
c.448T>C	p.Phe150Leu	—	23.5	VUS	Exon 2	Missense	[30]
c.453G>A	p.Val151Val	rs386134194	15.15	LP	Exon 2	Synonymous	[6, 21, 35]
c.457delTG	p.V153fsX193	—			Exon 2	Frameshift	[35]
c.458_459del	p.Val153Alafs*41	rs386134195	32	P	Exon 2	Frameshift	[6, 21]
c.470C>T	p.Ser157Phe	rs759925126	25.7	LP	Exon 2	Missense	[47]
c.494A>G	p.Asp165Gly	rs1449690838	28.7	VUS	Exon 2	Missense	[20]
c.495C>A	p.Asp165Glu	—	29.3	VUS	Exon 2	Missense	[50, 66, 91]
c.497+1G>T	—	—	34	P	Intron 2	Intron	[20, 24, 47, 59, 65]
c.498‐2A>G	—	rs1442518296	34	LP	Intron 1	Intron	[49]
c.505C>T	p.Arg169Trp	rs121908890	32	P	Exon 3	Missense	[6, 27, 35, 42, 57, 59, 62]
c.506G>A	p.Arg169Gln	rs121908889	32	P	Exon 3	Missense	[6, 21, 27, 31, 35, 45, 105]
c.506G>C	p.Arg169Pro	rs121908889	33	P	Exon 3	Missense	[6]
c.506G>T	p.Arg169Leu	—	33	LP	Exon 3	Missense	[49, 106]
c.507delG	p.K170Rfs*6	—	33	LP	Exon 3	Frameshift	[49]
c.517delC	p.Leu173CysfsTer3	—	33	LP	Exon 3	Frameshift	[29, 40, 57, 58, 59]
c.51C>G	p.Phe17Leu	rs11568520	25.5	P	Exon 1	Missense	[20, 22, 26, 27, 28, 34, 38, 40, 48, 57, 62, 65, 74, 76, 107, 108]
c.523G>A	p.Val175Met	rs781721860	18.18	VUS	Exon 3	Missense	[21, 101]
c.529A>G	p.Met177Val	rs145068530	22.6	LP	Exon 3	Missense	[27]
c.538C>G	p.Gln180Glu	rs1437174685	24.5	VUS	Exon 3	Missense	[49]
c.538C>T	p.Gln180X	—	42	LP	Exon 3	Nonsense	[82]
c.557T>C	p.Leu186Pro	rs386134197	24.4	LP	Exon 3	Missense	[6, 27]
c.565_568del	p.Phe189Argfs*14	—	33	LP	Exon 3	Frameshift	[6, 21]
c.56G>C	p.Arg19Pro	rs72552723	28.6	P	Exon 1	Missense	[21, 35, 63]
c.572A>G	p.Lys191Arg	rs200290813	12.4	LB	Exon 3	Missense	[41]
c.573delG	p.Asn192IlefsTer12	—	21.9	LP	Exon 3	Frameshift	[27]
c.573G>T	p.Lys191Asn	rs765204844	10.38	LB	Exon 3	Missense	[101]
c.597_597delG	p.Phe200Leufs*4	—	25.7	LP	Exon 3	Frameshift	[109]
c.610G>A	p.Gly204Ser	—	32	LP	Exon 3	Missense	[30]
c.621G>T	p.Gln207His	—	22.9	VUS	Exon 3	Missense	[20, 47, 110, 111]
c.629A>G	p.Asn210Ser	rs386134198	22.9	P	Exon 3	Missense	[6, 21, 112]
c.64_66del	p.Phe22del	rs377767444	41	P	Exon 1	Missense	[6, 21]
c.640_641delinsTT	p.Ala214Leu	—	28.3	LP	Exon 3	Missense	[30]
c.641C>T	p.Ala214Val	rs386134199	31	LP	Exon 3	Missense	[6, 21, 27, 35, 49, 62, 113]
c.652+1G>A	—	rs386134200	35	P	Intron 3	Intron	[44, 49, 65]
c.652+6G>A	—	rs4551059	18.51	VUS	Intron 3	Intron	[68]
c.653‐8T>A	—	—	15.8	VUS	Intron 3	Intron	[47]
c.64_66delTTC	p.Phe23del	rs377767444	21.7	P	Exon 1	Frameshift	[114, 115, 116]
c.688T>C	p.Phe230Leu	rs756650860	28.7	P	Exon 4	Missense	[27]
c.694A>G	p.Thr232Ala	rs188698686	28.5	LP	Exon 4	Missense	[103]
c.700G>C	p.Gly234Arg	rs1457258524	31	LP	Exon 4	Missense	[26, 44]
c.706T>C	p.Cys236Arg	rs747050292	27.6	LP	Exon 4	Missense	[45]
c.707G>A	p.Cys236Tyr	—	27.5	VUS	Exon 4	Missense	[30]
c.718G>A	p.Ala240Thr	—	22.9	LP	Exon 4	Missense	[27]
c.725G>T	p.Gly242Val	rs72552728	31	P	Exon 4	Missense	[6, 42]
c.740C>G	p.Pro247Arg	—	29.1	P	Exon 4	Missense	[6, 21]
c.744_745insTCG	p.Leu248_Phe249insSer	—		LP	Exon 4	Frameshift	[50]
c.745_748delTTTG	p.Phe249LeufsTer14	—	32	LP	Exon 4	Frameshift	[59]
c.752A>G	p.Tyr251Cys	—	27.3	VUS	Exon 4	Missense	[50, 66]
c.760C>T	p.Arg254Ter	rs121908893	35	P	Exon 4	Nonsense	[6, 20, 23, 30, 47, 48, 49, 50, 74, 99, 103, 108, 117, 118]
c.761G>A	p.Arg254Gln	rs200699819	32	P	Exon 4	Missense	[38, 46]
c.768G>A	p.Trp256Ter	rs386134202	40	LP	Exon 4	Nonsense	[6, 21, 55]
c.769C>T	p.Arg257Trp	rs386134203	26.4	LP	Exon 4	Missense	[27]
c.774_775insTCG	p.Met258_Leu259insSer	—	18.1	LP	Exon 4	Frameshift	[91]
c.77G>A	p.Ser26Asn	rs772578415	23.9	P	Exon 1	Missense	[6, 35]
c.782_799del	p.Val261_Pro266del	—	21.4	LP	Exon 4	Frameshift	[34, 47, 50, 51]
c.‐78C>T	—	rs13180043	7.875	B	5 prime UTR	UTR	[68]
c.791C>G	p.Thr264Arg	rs201262157	25.9	P	Exon 4	Missense	[27, 96]
c.791C>T	p.Thr264Met	rs201262157	24.6	LP	Exon 4	Missense	[27, 96]
c.797C>T	p.Pro266Leu	rs538372785	26.2	P	Exon 4	Missense	[30, 38, 46, 49, 50, 51]
c.806delT	p.Leu269Argfs*26	rs386134204	32	P	Exon 4	Frameshift	[6, 27]
c.807A>G	p.Leu269=	rs274558	7.089	B	Exon 4	Synonymous	[76]
c.822G>A	p.Trp274Ter	—	47	LP	Exon 4	Nonsense	[27, 34, 50, 51]
c.824+1G>A	—	rs1417000465	35	P	Intron 4	Intron	[49, 91]
c.825‐1G>C	—	rs1057516805	35	P	Intron 4	Intron	[30]
c.825‐52G>A	—	rs1194929977	0.591	LP	Intron 4	Intron	[71]
c.825G>A	p.Trp275Ter	rs386134207	47	P	Exon 5	Nonsense	[6, 21, 48, 62, 118]
c.835G>T	p.Glu279Ter	rs2126785892	35	LP	Exon 5	Nonsense	[53]
c.839C>T	p.Ser280Phe	rs386134208	31	P	Exon 5	Missense	[6, 21, 27, 34, 35, 39, 57, 119]
c.83G>T	p.Ser28Ile	rs72552724	28.9	P	Exon 1	Missense	[32]
c.844C>T	p.Arg282Ter	rs121908886	41	P	Exon 5	Nonsense	[6, 20, 21, 27, 28, 30, 32, 34, 35, 36, 50, 55, 104]
c.845G>A	p.Arg282Gln	rs386134210	34	P	Exon 5	Missense	[21, 24, 27, 34, 35, 54]
c.847T>A	p.Trp283Arg	rs72552729	32	P	Exon 5	Missense	[6, 21, 35, 55]
c.851T>C	p.Leu284Pro	—	29.6	LP	Exon 5	Missense	[49]
c.865C>T	p.Arg289Ter	rs386134212	35	P	Exon 5	Nonsense	[6, 21, 46, 49, 59]
c.895delA	p.K299Rfs*22	—	27.4	LP	Exon 5	Frameshift	[49]
c.902C>A	p.Ala301Asp	rs72552730	28.9	P	Exon 5	Missense	[21, 42]
c.‐91_22del	—	rs1554085861	—	LP	Exon 1	Start loss	[104]
c.919delG	p.Val307Leufs*15	—	22.4	LP	Exon 5	Frameshift	[29, 57]
c.92C>T	p.Pro31Leu	—	26.1	LP	Exon 1	Missense	[46]
c.934A>G	p.Ile312Val	rs77300588	16.57	LP	Exon 5	Missense	[27, 96]
c.949G>A	p.Glu317Lys	rs774792831	32	VUS	Exon 5	Missense	[96]
c.955C>T	p.Gln319Ter	rs1276208853	36	LP	Exon 6	Nonsense	[27]
c.95A>G	p.Asn32Ser	rs72552725	22.7	P	Exon 1	Missense	[6, 27, 30, 35, 36, 46, 54, 62, 71, 120, 121, 122]
c.976C>T	p.Gln326Ter	rs1047810495	36	P	Exon 6	Nonsense	[29, 57]
c.394‐16T>A	—	rs775097754	15.7	LP	Intron 1	Intron	[35]
c.820A>G	p.Met274Val	—	7.089	B	Exon 4	Missense	[76]
c.424G>T	p.Ala142Ser	rs151231558	21.3	LP	Exon 2	Missense	[27, 35, 62, 97, 123]
c.‐77G>A	—	rs13180295	7.598	B	5 prime UTR	UTR	[68]
c.535A>T	p.Met179Leu	rs386134196	17.43	VUS	Exon 3	Missense	[124]
c.1009delA	p.Thr337ProfsTer12	rs386134213	33	LP	Exon 6	Frameshift	[125]
c.849G>T	p.Trp283Cys	rs386134211	35	LP	Exon 5	Missense	[77]
c.1412G>A	p.Arg471His	rs386134223	33	P	Exon 8	Missense	[48, 59, 126]
c.833dup	p.Pro279Serfs*17	—	33	LP		Frameshift	[53]
c.248G>T	p.Arg83Leu	rs72552726	31	P	Exon 1	Missense	[6, 20, 21, 27, 35, 54, 83]
c.1027del	p.Val343Serfs*6	NA		LP	Exon 6	Frameshift	[127]
c.1441G>T	p.Val481Phe	rs11568513	13.58	VUS	Exon 8	Missense	[76]
c.53+20856A>T	NA	rs460407	2	B	IVS1	Intronic	[35]
c.395G>A	p.Trp132*	rs886041277	52	P	Exon 2	Nonsense	[94]
c.1131C>G	p.Phe377Leu	NA	19.10	VUS	Exon 7	Missense	[20]
c.1159T>C	p.Tyr387His	NA	23.6	VUS	Exon 7	Missense	[62]
c.1198C>T	p.Arg400Cys	rs1249681027	32	VUS	Exon 7	Missense	[128]
c.1199G>A	p.Arg400His	rs371219688	28.5	VUS	Exon 7	Missense	[20]
c.1229G>A	p.Gly410Asp	rs200125400	29.9	VUS	Exon 7	Missense	[129]
c.125T>C	p.Leu42Pro	NA	24.4	LP	Exon 1	Missense	[130]
c.1304delG	p.Gly435Alafs*24	rs386134217		P	Exon 8	Frameshift	[27]
c.1309T>C	p.Phe437Leu	NA	24.6	LP	Exon 8	Missense	[6]
c.1347del	p.Tyr449*	NA		P	Exon 8	Deletion	[48]
c.1361A>G	p.Val430Gly	NA	16.66	VUS	Exon 8	Missense	[6]
c.136C>G	p.Pro46Ala	rs202088921	25.6	LP	Exon 1	Missense	[54]
c.1392_1400delinsCA	p.Val465Thrfs*32	NA		LP	Exon 8	Frameshift	[6]
c.1449_1451insCA	p.Gly484Glnfs*16	NA		LP	Exon 8	Frameshift	[6]
c.144C>G	p.His48Gln	NA	25	LP	Exon 1	Missense	[21]
c.1489A>G	p.Ser497Gly	NA	23	VUS	Exon 9	Missense	[6]
c.1583_1584dup	p.Gly529Lysfs*3	NA		LP	Exon 9	Frameshift	[48]
c.186G>A	p.Trp62*	NA	44	LP	Exon 1	Nonsense	[90]
c.235del	p.His79Thrfs*51	rs377767447		P	Exon 1	Frameshift	[131]
c.252C>T	p.Tyr84=	rs1253026669	11.58	LB	Exon 1	Synonymous	[34]
c.255_256ins GCTCGCCACCG	p.Leu86Alafs*48	NA		LP	Exon 1	Frameshift	[132]
c.290T>C	p.Leu97Pro	NA	26.5	VUS	Exon 1	Missense	[34]
c.384dupT	p.Val129Cysfs*9	NA		P	Exon 1	Frameshift	[20]
c.393+1G>A	NA	rs1057517069	33	P	IVS1	Splicing	[95]
c.415G>A	p.Asp139Asn	rs577131769	24	VUS	Exon 2	Missense	[26]
c.422A>C	p.Lys141Thr	NA	26	VUS	Exon 2	Missense	[26]
c.447C>G	p.Phe149Leu	rs780989844	20	VUS	Exon 2	Missense	[62]
c.456_459del	p.Val153Cysfs*22	NA		LP	Exon 2	Frameshift	[6]
c.467G>C	p.Gly156Ala	NA	26.8	VUS	Exon 2	Missense	[31]
c.500T>C	p.Phe167Ser	NA	27	VUS	Exon 3	Missense	[21]
c.539A>T	p.Gln180Leu	NA	24.8	LP	Exon 3	Missense	[114]
c.653‐2A>C	NA	rs386134201	34	P	IVS3	Splicing	[21]
c.655_656del	p.Thr219Argfs*60	NA		LP	Exon 4	Frameshift	[6]
c.660_664del	p.Glu220Aspfs*58	NA		LP	Exon 4	Frameshift	[6]
c.697T>C	p.Leu233=	NA		VUS	Exon 4	Synonymous	[27]
c.725G>A	p.Gly242Asp	NA	34	LP	Exon 4	Missense	[21]
c.740C>T	p.Pro247Leu	NA	32	LP	Exon 4	Missense	[6]
c.745_746insCGT	p.Leu248_Phe249insSer	NA		LP	Exon 4	Insertion	[66]
c.768G>T	p.Trp256Cys	NA	33	LP	Exon 4	Missense	[21]
c.797C>T	p.Pro266Leu	rs538372785	27.6	P	Exon 4	Missense	[46, 91, 133]
c.821G>A	p.Trp274*	NA	45	P	Exon 4	Nonsense	[34]
c.845G>T	p.Arg282Leu	NA	34	LP	Exon 5	Missense	[21]
c.894C>A	p.Arg298=	NA	9	VUS	Exon 5	Synonymous	[27]
c.89delT	p.Ile30Thrfs*13	NA		LP	Exon 1	Frameshift	[24]
c.951+2T>C	NA	NA	27	LP	IVS5	Splicing	[48]
c.1232G>T	p.Gly411Val	NA	29.9	VUS	Exon 7	Missense	[125]
c.454G>C	p.Gly152Arg	NA	29	LP	Exon 2	Missense	[125]

*Note:* *Means: frameshift.

## 4 Discussion

PCD is an autosomal recessive disorder mainly caused by mutations in the *SLC22A5* gene, which encodes the organic cation transporter 2 (OCTN2). This transporter is essential for carnitine reabsorption in the kidneys and carnitine uptake in various tissues, including the heart and muscles [13, 22, 134]. The disorder typically manifests with a spectrum of clinical symptoms, ranging from fatigue to severe cardiomyopathy, highlighting the significant genetic contribution to disease pathology. More than 250 variants have been identified in *SLC22A5*, underscoring the gene's critical role in maintaining systemic carnitine levels [18, 135]. This metabolic disruption is associated with a high risk of developing cardiomyopathy, as the heart relies heavily on fatty acid metabolism for energy. Some common variants, including missense and nonsense variants, are found in exons 1 and 6 of the gene, representing key mutational hotspots that disrupt the normal function of OCTN2 [13, 18].

Carnitine availability, regulated by SLC22A5, is essential for mitochondrial import of long‐chain fatty acids via CPT‐I. Experimental inhibition of CPT‐I has been shown to confer cardioprotection by shifting myocardial substrate metabolism toward glucose utilisation. These findings support a mechanistic link whereby impaired carnitine transport may modify β‐oxidation rates, contributing to structural and functional cardiac changes observed in primary carnitine deficiency [136, 137]. Our results should therefore be interpreted not only in the context of genetic diagnosis but also within broader metabolic remodelling pathways.

### 4.1 Functional Characterisation of Identified Variants

The functional consequences of *SLC22A5* variants have been well documented through in vitro and in vivo studies, shedding light on how these mutations disrupt carnitine transport and cause cardiomyopathy. Mutations in the *SLC22A5* gene often result in the production of a dysfunctional OCTN2 protein, thereby directly impairing its ability to mediate carnitine uptake. This impairment reduces carnitine levels in cardiac and skeletal muscle, where fatty acid oxidation is crucial for energy production, thereby contributing to cardiomyopathy [6, 18, 135].

The correlation between specific *SLC22A5* variants and clinical manifestations of carnitine deficiency, particularly cardiomyopathy, has been explored in multiple studies. The clinical phenotype can vary widely depending on the nature of the mutation and its location, ranging from asymptomatic individuals to those with severe cardiac dysfunction. Nonsense mutations, which introduce premature stop codons, lead to truncated proteins that lack functional domains essential for carnitine transport, rendering the protein non‐functional [6, 134, 138]. It seems that among the variants, nonsense or frameshift mutations tend to have a greater effect on the OCTN2 protein's function. In contrast, missense mutations often lead to milder clinical presentations. However, this can vary depending on the specific amino acid change and its location within the protein structure [6, 15]. Frameshift mutations also have profound effects on OCTN2 function by altering the reading frame, leading to the production of aberrant proteins incapable of carnitine transport.

Genotype–phenotype correlations are critical for understanding how mutations in *SLC22A5* contribute to the spectrum of disease severity, particularly in cardiac complications [6, 13]. Patients with variants that preserve some transporter activity often present with delayed‐onset cardiomyopathy or milder symptoms, suggesting genotype‐based stratification to predict clinical outcomes. This highlights the importance of precise genetic screening and functional studies to guide prognosis and treatment decisions [15, 139].

Recent studies using mutagenesis assays and functional genomics approaches have enabled researchers to characterise the deleterious nature of individual variants, thereby improving our understanding of their role in disease progression. The identification of novel variants and their corresponding functional assays remains crucial for stratifying patients according to disease severity and optimizing therapeutic interventions [13, 134].

### 4.2 Identification of Novel Variants and Their Clinical Significance

As genomic technologies advance, the identification of novel *SLC22A5* variants has provided more profound insight into the genetic landscape of carnitine deficiency‐induced cardiomyopathy. Many newly discovered mutations have expanded our understanding of the diverse genotypic profiles that contribute to the disease. Novel variants, particularly those located in previously unexplored regions of the gene or those involving rare amino acid substitutions, often pose challenges in determining their pathogenic significance without functional characterisation [6, 12]. These novel variants can be classified as VUS until their clinical impact is established through segregation analysis, functional assays, and comparisons with population data. In particular, the identification of new mutations in regions outside the common sites, such as exons 1 and 8, which are among the most frequent variant regions, and exons 5 and 6, which have the highest CADD scores, has shed light on the broader functional domains of the OCTN2 protein.

These findings highlight the need for continuous updates to mutation databases and comprehensive tables of pathogenic sites, as presented in this systematic review, which will serve as valuable resources for future research and clinical applications [6, 138, 140]. The clinical significance of these novel variants often depends on their effect on carnitine transport capacity. Functional studies of mutant OCTN2 proteins have shown that some novel missense mutations retain partial carnitine transport activity, resulting in milder clinical presentations. Other variants, particularly those leading to severe structural changes, have been linked to more aggressive cardiomyopathy phenotypes. Thus, the integration of genetic and clinical data is essential to translate these findings into meaningful clinical practice, particularly for patient stratification to enable early intervention [6, 12].

### 4.3 Statistical Description of Variant Effects on the Gene Function

The present study revealed that the distribution of variants across exons and introns provides essential insights into potential functional impacts. Exon 1 shows the highest frequency of exon variants at 56 (20.7%), with a median CADD score of 28.60, suggesting a substantial likelihood of functional impact. Exon 8 follows closely in frequency and shows a slightly higher mean CADD score of 27.95. Other significant exons include Exon 4 and Exon 7, with notable frequencies and mean CADD scores ranging from 28.90 to 29.70. Interestingly, Exons 5 and 6 have lower frequencies but the highest mean CADD scores (32 and 33), indicating potentially deleterious effects despite their rarity.

In this review of variants, intronic regions and untranslated regions (UTRs) showed relatively low variant frequencies overall, each contributing less than 2% of detected variants. The most frequently affected region was intron 1 (1.9%), followed by intron 4 (1.5%) and intron 3 (1.1%), while introns 2 and 7 were rare (0.4% each). Despite their low frequency, several intronic variants showed notably high predicted deleteriousness, with median CADD scores around or above 34 in introns 1, 2, and 4. In contrast, variants in intron 3 exhibited lower predicted impact (median 18.51), suggesting heterogeneity in potential functional relevance across intronic regions. Overall, these findings indicate that while intronic variants in *SLC22A5* are uncommon, certain regions may harbour variants with higher predicted biological significance.

Furthermore, nonsense variants have the highest pathogenicity and CADD scores, followed by frameshift and missense variants, which also demonstrate substantial pathogenic potential.

### 4.4 Disease Severity and Outcome Stratification Based on Genotypic Profiles

The severity of carnitine deficiency‐induced cardiomyopathy and patient outcomes are closely linked to the underlying *SLC22A5* genotypic profiles. Stratification based on genotypes allows clinicians to predict disease course, optimise management strategies, and tailor treatment plans. Mutations resulting in complete loss of function, such as nonsense or frameshift mutations, are generally associated with early‐onset cardiomyopathy, rapid disease progression, and worse outcomes. Conversely, missense mutations with partial retention of function tend to result in milder disease phenotypes with later onset and slower progression [13, 15]. Studies have demonstrated that patients with primarily severe nonsense mutations are more likely to develop significant cardiac dysfunction and may require aggressive therapeutic interventions, including early and sustained carnitine supplementation. In contrast, patients with milder genotypes, such as those carrying missense mutations outside the key transport domains of OCTN2, may exhibit less severe cardiac involvement and can be managed with routine monitoring and conservative treatments [13, 76, 138]. Moreover, genotype‐based stratification has significant implications for prognosis. Individuals with high‐risk genotypes often present with more profound carnitine deficiencies. They are at greater risk for recurrent cardiac events or metabolic crises, necessitating more frequent clinical follow‐up and monitoring. In contrast, those with low‐risk genotypes may have fewer clinical manifestations, allowing for less intensive management. This stratification is crucial for optimizing patient outcomes and ensuring that therapeutic interventions are proportionate to the genetic risk [13, 22].

### 4.5 Challenges and Limitations in Variant Identification and Interpretation

Despite advancements in sequencing technologies and variant databases, identifying and interpreting *SLC22A5* variants remains challenging. One of the primary obstacles is the high degree of variability in the clinical expression of carnitine deficiency, even among individuals with the same mutation. This phenotypic variability complicates the interpretation of newly identified variants and hinders the ability to predict clinical outcomes based solely on genetic information [8, 12]. A significant challenge in variant identification is distinguishing between benign polymorphisms and pathogenic mutations, especially when a variant's functional consequences are unclear. Many variants, particularly those classified as variants of uncertain significance (VUS), require extensive functional studies and population‐level analyses to determine their pathogenicity. Furthermore, the rarity of some *SLC22A5* mutations limits the availability of data for comprehensive functional validation, making it difficult to assess their impact on carnitine transport and cardiomyopathy risk accurately [8, 114, 141].

Another limitation is the incomplete understanding of the full spectrum of *SLC22A5* variants and their interactions with environmental factors. While known frequent variants have been extensively studied, mutations in less‐characterised regions of the gene may also contribute to disease but remain poorly understood due to a lack of functional assays and clinical correlation studies. Moreover, many currently available variant databases do not incorporate real‐time updates, leading to outdated or incomplete information for clinical use [8, 12, 141].

While several studies have suggested a potential association between some variants in *SLC22A5* and earlier onset or more severe clinical manifestations, including cardiomyopathy, the available evidence remains limited. Most reported data are derived from individual case reports or small, heterogeneous cohorts with variable clinical characterisation. Therefore, although these findings may indicate a possible trend, they should be interpreted with caution. The heterogeneity in study design, patient selection, and reporting standards limits the ability to draw definitive genotype–phenotype correlations. Larger, well‐characterised cohort studies are needed to better elucidate these relationships and to determine the clinical significance of specific variant types.

In addition, in interpreting the genotype–phenotype correlations of SLC22A5 variants, several confounding factors must be considered. First, the effect of modifier genes cannot be overlooked. Genetic variants in genes encoding proteins involved in fatty acid oxidation, mitochondrial transport, or carnitine homeostasis may modulate disease severity or clinical penetrance [142]. Second, environmental influences play a critical role. Dietary carnitine intake, metabolic stress, infection, and other environmental exposures can significantly modify the clinical presentation, even among patients carrying identical SLC22A5 variants [143, 144]. These confounding factors help explain the observed phenotypic heterogeneity and highlight the importance of integrating both genetic and environmental contexts when evaluating patients with primary carnitine deficiency.

Although often overlooked, intronic and non‐coding variants in SLC22A5 may have important functional implications. Such variants can disrupt canonical splice sites or create cryptic splice sites, leading to aberrant transcripts, and may also influence transcriptional regulation by affecting promoters, enhancers, or untranslated regions. While current functional data regarding these variants are scarce, their potential impact on SLC22A5 expression and carnitine transport should not be underestimated [145]. The limited understanding of these mechanisms underscores the need for future studies employing transcriptomic and functional assays to better elucidate the pathogenic contributions of intronic and regulatory variants.

Of course, in terms of limitations, we did not perform a formal study‐level RoB assessment due to the predominance of non‐comparative designs; results should be interpreted in light of ACMG/CADD indicators and the presence/absence of functional validation. It is important to note that the limited and heterogeneous availability of functional validation data poses a significant challenge for interpreting SLC22A5 variants. As a result, some variants remain classified as VUS, reflecting the current inability to conclusively establish genotype–phenotype correlations in the absence of robust functional evidence.

Additionally, quantitative pooling was not feasible due to substantial heterogeneity in study designs and outcomes and the risk of overlapping cohorts with case reports. We therefore emphasise the use of structured narrative synthesis and descriptive statistics.

Finally, this systematic review makes one of the key contributions, the comprehensive table we have curated, which compiles and summarises known pathogenic variants of the SLC22A5 gene from previous studies. This table serves as a valuable resource for researchers, clinicians, and geneticists by consolidating mutations identified across diverse populations and clinical contexts. By meticulously collating data from prior studies, including common variant sites like exons 1 and 8, high‐CADD‐score and functionally impaired regions such as exons 5 and 6, and less‐characterised regions of the gene, our work provides a detailed reference point for future investigations. This table allows researchers to cross‐reference their findings with previously reported variants, facilitating the identification of novel mutations or VUS. By offering this detailed resource, we aim to enhance the understanding of genotype–phenotype correlations and support further exploration into the genetic underpinnings of carnitine deficiency‐induced cardiomyopathy. Beyond this study's role as a comprehensive catalogue of SLC22A5 variants, this study provides several insights into the genotype–phenotype spectrum of primary carnitine deficiency.

## 5 Conclusion

This systematic review summarises the role of SLC22A5 gene variants in causing carnitine deficiency–related cardiomyopathy. It shows that mutations disrupt carnitine transport, leading to systemic deficiency and cardiac dysfunction. Variants are most frequently reported in exons 1 and 8, while those in exons 5 and 6 may have higher pathogenic potential based on CADD scores. The study emphasises the need for continued discovery and functional analysis of novel variants, as well as integrating genetic, clinical, and environmental data to improve diagnosis, risk stratification, and treatment. Overall, although SLC22A5 mutations present diagnostic and therapeutic challenges, ongoing research may enable more precise, personalised management, with future work needed to better define genotype–phenotype relationships and explore new therapies.

## Author Contributions

**Amir Ghaffari Jolfayi:** formal analysis (equal), investigation (equal), methodology (equal), resources (equal), software (equal), writing – original draft (equal). **Mahdieh Soveizi:** investigation (equal), methodology (equal), software (equal). **Niloofar Naderi:** formal analysis (equal), investigation (equal), methodology (equal), software (equal). **Amirali Soheili:** data curation (equal), investigation (equal), methodology (equal), validation (equal), writing – original draft (equal). **Maryam Pourirahim:** investigation (equal), methodology (equal), software (equal). **Leyla Abdolkarimi:** data curation (equal), investigation (equal), validation (equal). **Majid Maleki:** conceptualization (equal), data curation (equal), validation (equal), visualization (equal), writing – review and editing (equal). **Samira Kalayinia:** conceptualization (equal), data curation (equal), project administration (equal), supervision (equal), writing – review and editing (equal).

## Funding

The authors have nothing to report.

## Conflicts of Interest

The authors declare no conflicts of interest.

## Data Availability

The data that support the findings of this study are available in public databases (PubMed and Scopus).
